# Great Task Ahead

**Published:** 1920-03-06

**Authors:** 


**THE PUBLIC WELL-BEING**—(*continued*).

### Great Task Ahead.

### Appalling Male Health Statistics.

Last week reference was made in these pages to the comprehensive records of health which the National Service Medical Boards obtained concerning men of military age in this country. These records, which have been issued in a detailed Report, covering a period from November 1917 to October 1918, contain an enormous amount of information of a remarkable nature, and should play a large part in supporting all those who are endeavouring to stimulate progress towards better organisation of health and greater regard for prevention and cure. It is, how-

**THE PUBLIC WELL-BEING—(*continued*).**

ever, no use starting at the period of military age, and it is still more useless to endeavour to tackle seriously any incidence of ill-health disclosed in the later categories of military age. The whole thing may be reduced to what, after all, is the solid starting-ground of everything in the slightest way connected with public health—namely, the proper care of infants and children, and all the many-sided considerations centred on that fundamental fact.

In the period covered by this report there were two and a-half million men examined. Out of these, only one man in three was found to be of the normal standard of health and strength for his age, and one man in ten was judged to be totally and permanently unfit for any form of military service. These are grave facts. It is too much to say that the permanently unfit men are physically useless for civil occupation, as there are certainly many men who, rejected for Army service, are yet able to carry on in civil life with reasonable care. But at the same time, it is quite probable that a large proportion of them are little more than physical wrecks. The report says there is no evidence of racial degeneration on a large scale, but there is ample evidence to show the baneful effect of modern conditions of life upon physique. It is difficult to say, if we could get similar statistics about women, whether they would give the same unsatisfactory picture, but there is no obvious reason to suppose that men in the bulk are less healthy than women; the chances, in fact, seem rather to be in the other direction. The statistics show that the worst physical defects were most prevalent in the industrial areas, which seems natural enough, but a low standard of health is not only confined to the towns, for even farm labourers, whose occupation might at least be considered amongst the healthiest, though their physique at twenty years of age is excellent, become middle-aged at forty, and are old men before their time.

From all regions dealt with there comes consistent condemnations of the industrial conditions, especially among the young. Boys are set to work for long hours at too early an age in conditions which enfeeble their physique, and this, combined with bad housing and lack of proper recreation, sows the seeds of permanent ill-health. Over and over again the report urges the necessity of some form of organised physical culture, fuller facilities for healthy recreation, better housing, and a greater knowledge of domestic economy. It is obvious that there is a long way to go in the way of social regeneration before we can hope for any sound and permanent improvement in the national health. Curiously enough, miners, despite their underground work, are amongst the best specimens of healthy manhood, and the worst specimens include barbers, masseurs, Turkish-bath attendants, etc., which, in the London and South-Eastern region, show a higher percentage of all diseases than any other trade group. The seriousness of this point is, therefore, that all this latter group could hardly be placed in a more dangerously easy position for the spread of the diseases from which they suffer.

Youths of eighteen only 4 ft. 9 in. in height, with a chest measurement of 29 in., and a weight of only 84 lb., were examined by Boards in industrial regions, and the report says the presence of such dwarfed and ill-developed creatures can be attributable only to the conditions of life created by our industrial development, eminently calculated to cause racial deterioration, and to give every opportunity to the ravages of disease. As an example of the defects in housing, it is pointed out that in Leeds alone there are about sixty thousand back-to-back houses, with a minimum of comfort and sanitation. Phthisis is particularly rife. Speaking generally of the Yorkshire and East Midland region, it is asserted that if an analysis were made of the children born in the slum districts, the result would be so appalling that no Government would have the slightest difficulty in passing any measure which would remedy this evil. In the Lancashire and Cheshire districts, especially in the big cities, the list of disabilities is described as appalling. It is a tremendous task that confronts the Ministry of Health. It touches the national life at almost every point, and it will need great driving force both within the Administration and without if any real progress is to be achieved.

